# *Arachis batizocoi*: a study of its relationship to cultivated peanut (*A. hypogaea*) and its potential for introgression of wild genes into the peanut crop using induced allotetraploids

**DOI:** 10.1093/aob/mcu237

**Published:** 2014-12-20

**Authors:** Soraya C. M. Leal-Bertioli, Silvio P. Santos, Karinne M. Dantas, Peter W. Inglis, Stephan Nielen, Ana C. G. Araujo, Joseane P. Silva, Uiara Cavalcante, Patricia M. Guimarães, Ana Cristina M. Brasileiro, Noelia Carrasquilla-Garcia, R. Varma Penmetsa, Douglas Cook, Márcio C. Moretzsohn, David J. Bertioli

**Affiliations:** ^1^Embrapa Genetic Resources and Biotechnology, PqEB W5 Norte Final, CP 02372, CEP 70.770-917, Brasília, DF, Brazil, ^2^University of Brasilia, Institute of Biological Sciences, Campus Darcy Ribeiro, CEP 70.910-900, Brasília, DF, Brazil, ^3^Catholic University of Brasilia, Biotechnology and Genomic Sciences, SGAN 916 Avenida W5, CEP 70.790-160, Brasilia, DF, Brazil, ^4^Plant Breeding and Genetics Section, Joint FAO/IAEA Division, International Atomic Energy Agency, Vienna International Centre, Vienna A-1400, Austria and ^5^Department of Plant Pathology, University of California at Davis, One Shields Avenue, Davis, CA 95616, USA

**Keywords:** *Arachis batizocoi*, *A. hypogaea*, peanut, groundnut, pre-breeding, allotetraploid, polyploidization, introgression, wild species, GISH, orthologous genes, intron sequences, K genome

## Abstract

**Background and Aims** *Arachis batizocoi* is a wild relative of cultivated peanut (*A. hypogaea*), an allotetraploid with an AABB genome. *Arachis batizocoi* was once considered the ancestral donor of the peanut B genome, but cytogenetics and DNA phylogenies have indicated a new genome classification, ‘K’. These observations seem inconsistent with genetic studies and breeding that have shown that *A. batizocoi* can behave as a B genome.

**Methods** The genetic behaviour, genome composition and phylogenetic position of *A. batizocoi* were studied using controlled hybridizations, induced tetraploidy, whole-genome *in situ* fluorescent hybridization (GISH) and molecular phylogenetics.

**Key Results** Sterile diploid hybrids containing AK genomes were obtained using *A. batizocoi* and the A genome species *A. duranensis*, *A. stenosperma*, *A. correntina* or *A. villosa*. From these, three types of AAKK allotetraploids were obtained, each in multiple independent polyploidy events. Induced allotetraploids were vigorous and fertile, and were hybridized to *A. hypogaea* to produce *F*_1_ hybrids. Even with the same parental combination, fertility of these *F*_1_ hybrids varied greatly, suggesting the influence of stochastic genetic or epigenetic events. Interestingly, hybrids with *A. hypogaea* ssp. *hypogaea* were significantly more fertile than those with the subspecies *fastigiata*. GISH in cultivated × induced allotetraploids hybrids (harbouring AABK genomes) and a molecular phylogeny using 16 intron sequences showed that the K genome is distinct, but more closely related to the B than to the A genome.

**Conclusions** The K genome of *A. batizocoi* is more related to B than to the A genome, but is distinct. As such, when incorporated in an induced allotetraploid (AAKK) it can behave as a B genome in crosses with peanut. However, the fertility of hybrids and their progeny depends upon the compatibility of the A genome interactions. The genetic distinctness of *A. batizocoi* makes it an important source of allelic diversity in itself, especially in crosses involving *A. hypogaea* ssp. *hypogaea*.

## INTRODUCTION

The genus *Arachis* is endemic to South America, found over a wide area and in diverse environments. It contains 81 described species, assembled into nine taxonomical sections, according to their morphology, geographic distribution and cross-compatibility (Krapovickas and Gregory, 1994; Valls and Simpson, 2005; Valls *et al.*, 2013). Peanut and another 30 known wild species reside in the Section *Arachis*. Most species are diploid (2*n* = 2*x* = 20), three are aneuploid or dysploid (2*n* = 2*x* = 18) and two are allotetraploids (2*n* = 4*x* = 40). The allotetraploids, the wild *A. monticola* and the cultivated peanut *A. hypogaea* are very closely related and belong to the same biological species. Within and between these tetraploid species, DNA polymorphism is very low (Halward *et al.*, 1991; Kochert *et al.*, 1996), and it seems most likely that a single, or very few polyploidy events involving the same ancestors, gave rise to both species only a few thousand years ago (Bertioli *et al.*, 2011).

Peanut is a very important food crop throughout the tropics and sub-tropics, and its very narrow genetic base presents limitations for crop improvement. These limitations have long been apparent for breeding for pest and disease resistances, and there are strong reasons to believe that introducing allelic diversity from wild species could also improve more complex traits such as yield and drought response. Correspondingly, peanut breeders have been interested in introducing wild species into breeding programmes. However, the ploidy level difference and fertility barriers between peanut and its wild relatives hamper their effective use. Clearly, an understanding of the relationships of the cultivated and wild species would facilitate the production of fertile hybrids and, consequently, introgression of wild genes into the peanut crop. In this regard, many studies have focused on the species with 20, mostly metacentric chromosomes in the section *Arachis*, because these include cultivated peanut and its closest wild relatives (here we will not deal with the 2*n* = 2*x* = 18 or asymmetric karyotype species). These species were initially divided into two genome types, A and B, based on the component sub-genomes of *A. hypogaea*. The A genomes are characterized by the presence of a small pair of chromosomes with allocyclic condensation, ‘the A chromosomes’ (Husted, 1936; Smartt *et al.*, 1978), whereas the B genomes are characterized by the absence of these A chromosomes. The validity of the A genome grouping is strongly supported by diverse evidence. This includes their moderate to high cross-compatibility; unifying biological characteristics, such as predominantly perennial growth and chromosomes with condensed A/T-rich centromeres; and their grouping in molecular phylogenies. The definition of the B genome group is more precarious since it is based on the absence of a character (the small A chromosomes). Accordingly, further cytogenetic and molecular phylogenetic studies justified the sub-division of species traditionally assigned to this group: *A. ipaënsis*, which is now thought to be ancestral to *A. hypogaea* and its closest relatives remains the B genome [also known as the B genome *sensu stricto* (*s.s.*)], while *A. batizocoi* and two closely related species are assigned as a new grouping, the ‘K’ genome, and another two species are assigned to the F genome (Robledo and Seijo, 2010). The data available on chromosome behaviour during meiosis in diploid hybrids are consistent with the K genome being distinct from both the A and B genomes. Intragenome hybrids (A × A, B × B and K × K) generally show a mean bivalent formation of the chromosomes >9·5 out of the expected 10, whilst intergenome hybrids (A × B, A × K and B × K) show values <8 (Robledo and Seijo, 2010). Furthermore, with this revision and subsequent works, the phylogenetic position of the K genome relative to the A and B genomes in *Arachis* became a matter of uncertainty. A notable feature that distinguishes the K genome chromosomes is the presence of an A genome-like condensed centromere, but the absence of the ‘A chromosome’. Also, whilst some molecular phylogenetic studies placed the species assigned as K genome closer to the B genome, (Moretzsohn *et al.*, 2004; Bechara *et al.*, 2010; Friend *et al.*, 2010), others placed it closer to the A genome (Tallury *et al.*, 2005; Robledo *et al.*, 2009; Robledo and Seijo, 2010; Moretzsohn *et al.*, 2013).

The establishment of the K genome group sits uncomfortably with the results from a series of classic experiments, which were started when *A. batizocoi* was considered the B genome donor to the cultivated species (Smartt *et al.*, 1978; Stalker *et al.*, 1991). Using a hybridization scheme known as ‘the tetraploid route’, Simpson *et al.* (1993) obtained a wild-derived allotetraploid (also referred to as amphidiploid). To do this, an A genome diploid (in this case, a hybrid between *A. cardenasii* and *A. diogoi*) was crossed with *A. batizocoi*, giving origin to a sterile hybrid. This was treated with colchicine to induce chromosome doubling and restore fertility. The resultant allotetraploid [*A. batizocoi* × (*A. cardenasii* × *A. diogoi*)]^4×^ was registered as TxAG-6 and produced viable hybrids when crossed with cultivated peanut. A *BC*_1_ population derived from the peanut cultivar Florunner and TxAG-6 was used to generate the first peanut tetraploid map (Burow *et al.*, 2001). This map showed that the genome of *A. batizocoi* recombined with the B genome of *A. hypogaea* in a relatively regular mode. Furthermore, successive backcrossing followed by selection allowed the development of a number of important cultivars such as COAN, NemaTAM and Tifguard (Simpson and Starr, 2001; Simpson *et al.*, 2003; Holbrook *et al.*, 2008), which harbour a chromosome segment from *A. cardenasii* conferring root-knot nematode resistance (Nagy *et al.*, 2010).

Here we used controlled hybridizations, induced polyploidy, whole-genome *in situ* fluorescent hybridization (GISH) and the construction of a molecular phylogeny to better define the K genome of *A. batizocoi* and its potential for use in hybridization schemes designed to introduce wild alleles into the peanut crop.

## MATERIALS AND METHODS

### Production of hybrids and induced allotetraploids

*Arachis* seeds were obtained from the Active Germplasm Bank of Embrapa Genetic Resources and Biotechnology (Cenargen, Brasília, Brazil). Plants were grown in an open-plan greenhouse. Diploid wild genotypes used for crosses are listed in Table 1. *Arachis batizocoi* was used as female parent and A genome accessions were used as male parents. All crosses were performed essentially as described by Simpson (1991), between the months of October and February.

**Table 1.** Wild and cultivated *Arachis* genotypes used for crosses; induced allotetraploids and *F*_1_ hybrids were produced in this study

Genotype	Plant ID	Ploidy	Genome type	Collection site/classification
Wild diploid species				
*A. batizocoi* Krapov. & W.C. Gregory	K9484	2*x*	KK	Parape, Bolívia
*A. batizocoi* Krapov. & W.C. Gregory	GKBSPSc30081*	2*x*	KK	Lagunillas, Bolívia
*A. correntina* (Burkart) Krapov. & W.C. Gregory	GKP9548	2*x*	AA	Corrientes, Argentina
*A. duranensis* Krapov. & W.C. Gregory	V14167	2*x*	AA	Salta, Argentina
*A. duranensis* Krapov. & W.C. Gregory	SeSn2848	2*x*	AA	Salta, Argentina
*A. duranensis* Krapov. & W.C. Gregory	KSSc3603*	2*x*	AA	Salta, Argentina
*A. duranensis* Krapov. & W.C. Gregory	WiSVg1510-B*	2*x*	AA	Boquerón, Paraguay
*A. ipaënsis* Krapov. & W.C. Gregory	KG30076	2*x*	BB	Gran Chaco, Bolivia
*A. stenosperma* Krapov. & W.C. Gregory	V13828	2*x*	AA	Luis Alves, GO, Brazil
*A. stenosperma* Krapov. & W.C. Gregory	V10309	2*x*	AA	Rondonopolis, MT, Brazil
*A. stenosperma* Krapov. & W.C. Gregory	V7762	2*x*	AA	Araguaiana, MT, Brazil
*A. villosa* Benth	V12812	2*x*	AA	Bella Unión, Uruguay
Peanut accessions				
*A. hypogaea* subsp. *fastigiata* var. *fastigiata* L.	BR1	4*x*	AABB	Modern cultivar
*A. hypogaea* subsp. *fastigiata* var. *fastigiata* L.	55437 (Senegal)	4*x*	AABB	Modern cultivar
*A. hypogaea* subsp. *fastigiata* var. *fastigiata* L.	BRS-Havana	4*x*	AABB	Modern cultivar
*A. hypogaea* subsp. *hypogaea* var. *hypogaea* L.	IAC-Caiapó	4*x*	AABB	Modern cultivar
*A. hypogaea* subsp. *hypogaea* var. *hypogaea* L.	Runner IAC 886	4*x*	AABB	Modern cultivar
Induced allotetraploids				
[*A. batizocoi* K9484 × *A. duranensis* V14167]^4×^	BatDur1	4*x*	AAKK	Synthetic alotetraploid
[*A. batizocoi* K9484 × *A. duranensis* SeSn2848]^4×^	BatDur2	4*x*	AAKK	Synthetic alotetraploid
[*A. batizocoi* K9484 × *A. stenosperma* V10309]^4×^	BatSten1	4*x*	AAKK	Synthetic alotetraploid
*F*_1_ hybrids between induced allotetraploids and peanut				
IAC-Caiapó × [*A. batizocoi* K9484 × *A. duranensis* V14167]^4×^	Caiapo × BatDur1	4*x*	AABK	*F*_1_ hybrid
IAC-Caiapó × [*A. batizocoi* K9484 × *A. duranensis* SeSn2848]^4×^	Caiapo × BatDur2	4*x*	AABK	*F*_1_ hybrid
IAC-Caiapó × [*A. batizocoi* K9484 × *A. stenosperma* V10309]^4×^	Caiapo × BatSten1	4*x*	AABK	*F*_1_ hybrid
Runner IAC 886 × [*A. batizocoi* K9484 × *A. duranensis* V14167]^4×^	Runner × BatDur1	4*x*	AABK	*F*_1_ hybrid
Runner IAC 886 × [*A. batizocoi* K9484 × *A. duranensis* SeSn2848]^4×^	Runner × BatDur2	4*x*	AABK	*F*_1_ hybrid
Runner IAC 886 × [*A. batizocoi* K9484 × *A. stenosperma* V10309]^4×^	Runner × BatSten1	4*x*	AABK	*F*_1_ hybrid
Runner IAC 886 × [*A. ipaënsis* KG30076 × *A. duranensis* V14167]^4×^	Runner × IpaDur1	4*x*	AABB	*F*_1_ hybrid
BR1 × [*A. batizocoi* K9484 × *A. duranensis* V14167]^4×^	BR1 × BatDur1	4*x*	AABK	*F*_1_ hybrid
BR1 × [*A. batizocoi* K9484 × *A. duranensis* SeSn2848]^4×^	BR1 × BatDur2	4*x*	AABK	*F*_1_ hybrid
BR1 × [*A. batizocoi* K9484 × *A. stenosperma* V10309]^4×^	BR1 × BatSten1	4*x*	AABK	*F*_1_ hybrid
BRS Havana × [*A. batizocoi* K9484 × *A. duranensis* V14167]^4×^	Havana × BatDur1	4*x*	AABK	*F*_1_ hybrid
BRS Havana × [*A. batizocoi* K9484 × *A. duranensis* SeSn2848]^4×^	Havana × BatDur2	4*x*	AABK	*F*_1_ hybrid
BRS Havana × [*A. batizocoi* K9484 × *A. stenosperma* V10309]^4×^	Havana × BatSten1	4*x*	AABK	*F*_1_ hybrid
55-437 × [*A. batizocoi* K9484 × *A. duranensis* V14167]^4×^	55-437 × BatDur1	4*x*	AABK	*F*_1_ hybrid
55-437 × [*A. batizocoi* K9484 × *A. duranensis* SeSn2848]^4×^	55-437 × BatDur2	4*x*	AABK	*F*_1_ hybrid
55-437 × [*A. batizocoi* K9484 × *A. stenosperma* V10309]^4×^	55-437 × BatSten1	4*x*	AABK	*F*_1_ hybrid

*Accessions used for phylogenetic analysis only.

Hybrid plants were initially identified by their aggregated pollen masses and absence of peg production. To confirm hybrid identity, marker analysis with the simple sequence repeat (SSR) Ah-041 (Moretzsohn *et al.*, 2004) was used. Genomic DNA was extracted from fresh, young leaves according to Ferreira and Grattapaglia (1998). PCRs were performed in a 13 µL volume: 1× PCR buffer (10 mm Tris–HCl pH 8·3, 50 mm KCl, 2·0 mm MgCl_2_), 0·2 mm of each dNTP, 1 U of *Taq* DNA polymerase (Invitrogen, USA), 1 ng µL^−1^ bovine serum albumin (BSA), 5 pmol of primer (Ah-041-Fwd, 5′-CGCCACAAGATTAACAAGCACC-3′; Ah-041-Rev, 5′-GCTGGGATCATTGTAGGGAAGG-3′) and 10 ng of genomic DNA. Amplifications were done in an ABI 9700 thermocycler (Applied Biosystems, Foster City, CA, USA) with the following cycling conditions: 96 °C for 2 min, followed by 30 cycles of 94 °C for 1 min, 60 °C for 1 min, 72 °C for 1 min and a final extension of 72 °C for 7 min. Fragments were separated in a 5 % polyacrylamide gel, and stained with silver nitrate (Creste *et al.*, 2001).

Confirmed hybrid plants (harbouring AK genomes) were treated with colchicine to induce chromosome doubling (Simpson, 1991). Cuttings from lateral branches were submerged in a solution of 0·2 % colchicine for 10 h. The cuttings were then rinsed in water for 15 min and planted in sand. Upon rooting and the appearance of new shoots, cuttings were transferred to a substrate containing 60 % sand and 40 % red clay-rich soil supplemented by 1500 g of NPK 4-30-16 per m^3^ of soil. The production of pegs and seeds signalled the recovery of fertility of some sectors in the colchicine-treated hybrids. Progeny from these treated hybrids were considered putative induced allotetraploids. Cytogenetic analysis (see below) was used to confirm their ploidy level and chromosome complement.

### Morphological measurements of induced allotetraploids and diploid parentals

Dimensions of seeds of allotetraploids and their diploid parents were determined using a digital caliper, at the longest and widest point of each seed. Ten fresh seeds/plant were measured and weighed. For plants that produced <10 seeds, all seeds available were evaluated. The first fully expanded leaf from the apex of at least eight different plants of each genotype were scanned and the areas were determined using the software QUANT® (Vale *et al.*, 2001). *Arachis hypogaea* cv. Tatu was included as control.

### Hybridization of *A. hypogaea* with induced allotetraploids

Five cultivars belonging to the two subspecies of peanut, *hypogaea* and *fastigiata*, were used as female parents in crosses with the obtained allotetraploids (Table 1). Crosses were performed between the months of February and June. To determine crossability of allotetraploids with cultivated peanut genotypes (the number of *F*_1_ hybrids produced per cross), pollinations and peg production were logged daily. Putative *F*_1_ hybrids were confirmed by the presence of yellow flower colour inherited from the allotetraploids used as male parents (the five cultivars used here have orange flowers). Pollen viability of *F*_1_ hybrids was estimated by the staining method with acetic carmine (Linsley and Cazier, 1963). For each genotype, 1000 pollen grains were analysed from oblong anthers as follows: 100 pollen grains per anther, two anthers per flower and five flowers per hybrid. Hybrid fertility was estimated by observation of peg production and by the number of seeds produced per *F*_1_ hybrid.

### Statistical analyses

All data were analysed using the statistical software R. To verify normality, the Shapiro–Wilk test was used. When data presented normality, the comparisons were performed using standard variance analyses, followed by the Tukey test of multiple comparisons. When data did not follow normal distribution, generalized linear models were used to relax assumptions about response variable and errors. Significance level in all analyses was 0·05. Average silhouette width, *s*(*i*), using K-means was used to create groupings (values vary between 0 and 1, and observations closer to 1 were better clustered).

### Cytogenetic analyses

Clean young root tips for cytogenetic analysis were produced by rooting the petioles of leaves in moist cotton wool in humid Petri dishes at about 25 °C in illuminated conditions. Root tips were treated with 2 mm 8-hydroxyquinoline for 2 h at room temperature and another hour on ice prior to fixation in ethanol:acetic acid solution (3:1, v/v) for 24 h at 4 °C. Fixed root tips were incubated in enzyme buffer (4 mm citric acid, 6 mm sodium citrate, pH 4·8) containing 2 % cellulose Onozuka R10 (Merck) and 20 % pectinase (Sigma) for 45 min at 37 °C. Chromosome spreads were made using a modified technique originally described by Pijnacker and Ferwerda (1984). Briefly, meristem cells of a single root tip were suspended in 15 µL of 60 % acetic acid on a microscope slide. The slide was heated to 45 °C, another drop of acetic acid was added and, after 1 min, the chromosome suspension was precipitated by adding fixative solution in excess.

Genomic DNAs for GISH consisted of sheared genomic DNA, labelled with either digoxigenin-11-dUTP or biotin-11-dUTP (Roche) by random primed labelling. Here, digoxigenin-labelled genomic DNA of *A. duranensis* and biotin-labelled genomic DNA of *A. ipaënsis* were used, both at 65 ng µL^−1^. Pre-treatment, hybridization, washing and detection procedures essentially followed the protocols of Schwarzacher and Heslop-Harrison (2000). Denatured DNA probes were mixed in a hybridization solution containing 50 % (v/v) formamide, 10 % (w/v) dextran sulphate, 0·125 mm EDTA, 0·125 % (w/v) SDS and 1 µg of salmon sperm DNA. The chromosomes and hybridization mix were denatured together at 81·5 °C for 10 min before hybridization overnight at 37 °C. Post-hybridization washes were carried out at 83 % stringency. Hybridization sites were detected by sheep anti-digoxigenin conjugated to fluorescein isothiocyanate (antibody anti-dig conjugated to FITC; green fluorescence) and Cy3–streptavidin (red fluorescence). FITC signals were amplified using anti-sheep antibody conjugated to fluorescein (Vector Laboratories, USA). Chromosomes were counterstained with DAPI (4',6-diamidino-2-phenylindole; blue fluorescence). Slides were observed in a Zeiss Axiophot epifluorescence microscope using appropriate filters. Images were captured and analysed using the CCD camera AxioCam MRm in combination with the AxioVison software (both Zeiss, Germany) and processed in Adobe Photoshop CS.

### Phlyogenetic analysis of A, B and K genomes

#### Intron sequencing.

In order to better define the phylogenetic position of *A. batizocoi* with respect to the *Arachis* A and B *s.s.* genomes, we used orthologous sequences from three accessions of *A. duranensis* (A genome; accession nos K7988, KSSc3603 and WiSVg1510-B), one of *A. stenosperma* (A genome; V10309), two of *A. batizocoi* (K genome, *sensu lato* B genome; K9484 and GKBSPSc30081), one of *A. ipaënsis* (B genome *s.s.*; KG30076) and one from *A. magna* (B genome *s.s.*; KG30097). Orthologous coding regions tend to be highly conserved and thus phylogenetically uninformative in closely related species. Therefore we used orthologous intron sequences, amplifying them via PCR primers complementary to conserved exon sequences that flank the introns (Choi *et al.*, 2006; Penmetsa and Cook, unpubl. res.). Primer pairs designed to amplify the 16 tentative orthologous genes (TOGs) are described in Table 2. Sequencing was performed using the BigDye 3.1 Terminator sequencing kit (Applied Biosystems) on ABI sequencers (Applied Biosystems). Sequences were processed using the Staden Package (Staden, 1996), with base calling using Phred (Staden, 1996; Ewing and Green, 1998).

**Table 2.** Primer pairs used for amplication of Sanger-sequence tentative orthologous genes (TOGs)

TOG amplicon	F oligo sequence (5′ > 3′)	R oligo sequence (5′ > 3′)
TOG896852	GGYTCCTTGCAAATGTTCRT	GAGGCCAATAAAGCCATTCA
TOG896966	GTGCTCTTCCCATTTCRGTA	TCCATGGTTTTGCTTCATCA
TOG897172	AGCATATCATTRGAGCCWGG	CACTTTGGCTGGAAACCAAT
TOG897242	GATGGGATCCSAAGCAACTW	GRGTATGCCCAAGAAGTTCM
TOG897648	CGATSYTGTGGGAGAACATT	WGGAGCTTCACAAATGCAAA
TOG898975	AARGCTCTTGRCACCTTTCA	GGACCGGGACAGACMACRGT
TOG899382	GTCTTGGAGAAGACCGCTTG	CATCATGCACAAGTTCCTKG
TOG899751	GTTGACCTGCACCCTYTGYT	TTCCATTGCAATAGCATCCA
TOG900140	GGCMCATGTTGTTATGGCTG	TTCCAWGCTTCAGCAAACCT
TOG900220	CATTTGCWTTAGCTGCCTCA	CCATGCTGAGWACTTGCGAA
TOG901841	WTGCATCATTGGGSAATCTA	AAGGTCCCATATGTTYGCAS
TOG903928	GACTGTTCGCCAYCATGCTR	CCTTTGCTRCTTTCCCACCR
TOG904180	CGTGCCAAAGCTGTRCTTSA	GGTGTTCAAGAARCGACCMC
TOG908826	TCCRGGTGAAGGTTGTTAAG	CTGGTTGTCTGCAGATTGGA
TOG910656	GATTGCATCCTGATGGTYCT	CAACACGGGAGCCTACRGAT
TOG923111	TAGCAGTGGTGGTGGTCAAA	CWGGYCCACCAAAGAGATAA

#### Sequence alignment and phylogenetic analysis.

*Arachis* intron sequences were aligned using Muscle (Edgar, 2004), and concatenated in SequenceMatrix 1.7.8 (Vaidya *et al.*, 2011). Several of the TOG introns were found to be rich in indels, which were encoded and appended to the data matrix as C or A base transversions for presence or absence, respectively, using the simple coding scheme of Simmons and Ochoterena (2000), as implemented in the program Fastgap 1.2 (Borchsenius, F. 2009. FastGap 1.2. Department of Biosciences, Aarhus University, Denmark. Published online at http://www.aubot.dk/FastGap_home.htm).

A phylogenetic hypothesis based on the concatenated TOGs and encoded indel characters was obtained using the Bayesian Markov Chain Monte Carlo (MCMC) method as implemented in MrBayes 3.2.2 (Ronquist *et al.*, 2012). Reversible-jump MCMC (Huelsenbeck *et al.*, 2004) was invoked for model optimization within the GTR family (nst = mixed rates = invgamma), and priors allowed to vary independently for each of the 17 data partitions (each TOG + indel matrix). One cold and three heated MCMC chains were run in parallel for 2 million generations, sampling every 1000 generations. This run time was sufficient for the convergence diagnostic, the mean standard deviation of split frequencies, to fall to 0·003612. The first 25 % of the trees were discarded (the burn-in) prior to calculation of the 50 % majority rule consensus tree. Convergence and adequacy of the burn-in were confirmed by visualization of tree probabilities in the program Tracer v1.5 (Rambaut *et al.*, 2014), available from http://beast.bio.ed.ac.uk/Tracer, where a normal distribution of log likelihood values was obtained for both MCMC runs (data not shown).

#### Testing for ancient hybridization events in the Arachis species evolution.

To detect possible ancient hybridization events and reticulate evolution between the genomes of the included *Arachis* species, we analysed the aligned and concatenated matrix of the 16 TOGs and appended coded indel characters in SplitsTree 4 (Huson and Bryant, 2006), using the parsimony splits method. We also compared the results with a similar analysis where data from *A. hypogaea* ‘Runner IAC 886’ were included in the matrix as a recombination-positive control. The PHI statistical test (Bruen *et al.*, 2006) was also used to infer recombination along the concatenated alignment, with a window size of 100 bases. We also attempted to detect recombination using the Bayesian dual Multiple Change-Point (MCP) model, with a default starting window size of 200 bases, as implemented in the DualBrothers program (Minin *et al.*, 2005) within the Geneious environment (Drummond *et al.*, 2011).

## RESULTS

### Production of hybrids and artificially induced allotetraploids

Sixty-seven marker-confirmed diploid AK genome hybrid plants, representing seven different hybrid combinations, were generated with crosses between *A. batizocoi* and seven accessions containing the A genome of four different species (Table 3). All diploid hybrids had aggregated pollen masses and did not produce progeny. Five out of the seven combinations were vigorous, but two, *A. batizocoi* × *A. correntina* and *A. batizocoi* × *A. villosa*, showed altered morphological development. The former developed spontaneous tumour-like growth at the base of the stems, but otherwise grew vigorously. The latter developed spontaneous necrotic patches on the leaves, followed, when the plants grew older, by leaf drop and eventual death. These irregular phenotypes, indicative of hybrid incompatibilities, occurred with all independently obtained individuals of these hybrid combinations.

**Table 3.** Production of allotetraploid plants

Cross	N-hyb	N-allo	T/D
*A. batizocoi* K9484 × *A. correntina* GKP9548	18	0	0·00
*A. batizocoi* K9484 × *A. duranensis* V14167	12	23	1·92
*A. batizocoi* K9484 × *A. duranensis* SeSn2848	8	12	1·50
*A. batizocoi* K9484 × *A. stenosperma* V10309	9	31	3·44
*A. batizocoi* K9484 × *A. stenosperma* V13828	9	0	0·00
*A. batizocoi* K9484 × *A. stenosperma* V7762	7	0	0·00
*A. batizocoi* K9484 × *A. villosa* V12812	4	0	0·00
Total	67	66	

N-hyb, number of diploid hybrids; N-allo, number of induced allotetraploids obtained after colchicine treatment; T/D, allotetraploid/diploid hybrid ratio.

**Fig. 1.**
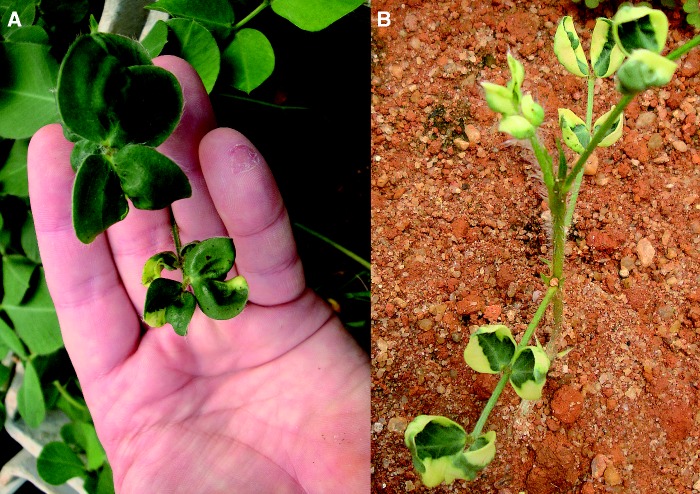
A leaf of [*A. batizocoi* K9484 × *A. duranensis* SeSn2848]^4×^ (BatDur2) as an example of the phenotypic abnormalities typical of young leaves of induced allotetraploids, especially in early generations. Note the bleached and distorted leaf lamina and necrotic spots that appear to be spontaneous (A) and a plantlet with heavily bleached leaves (B).

After treatment with colchicine, the stem cuttings showed some necrosis, delayed rooting and production of distorted leaves. Many died, but others successfully produced new plants. Of these, restoration of fertility was observed in three of the seven hybrid combinations. This was indicated by the production of gynophores and development of seeds. From these primary seeds, 66 independent fertile allotetraploid plants were produced. These induced allotetraploids plants combined the K genome of *A. batizocoi* K9484 with the A genome of *A. duranensis* V14167, *A. duranensis* SeSn2848 or *A. stenosperma* V10309. They were named BatDur1, BatDur2 and BatSten1, respectively (Table 3). Germinated allotetraploid seedlings usually presented developmental aberrations, such as the absence of chlorophyll in some leaflet areas, small curled distorted leaves and delimited sectors of necrosis in the young leaves (Fig. 1), but these characteristics disappeared about 3 weeks after germination. These characters reappeared in seedlings of subsequent generations, with an overall tendency of decreasing with each passing generation. Allotetraploid plants which originated from the same diploid parents, but from different polyploidy events, were phenotypically uniform, except for one white-flowered mutant of BatSten1.

### Morphological measurements of induced allotetraploids and diploid parentals

**Fig. 2.**
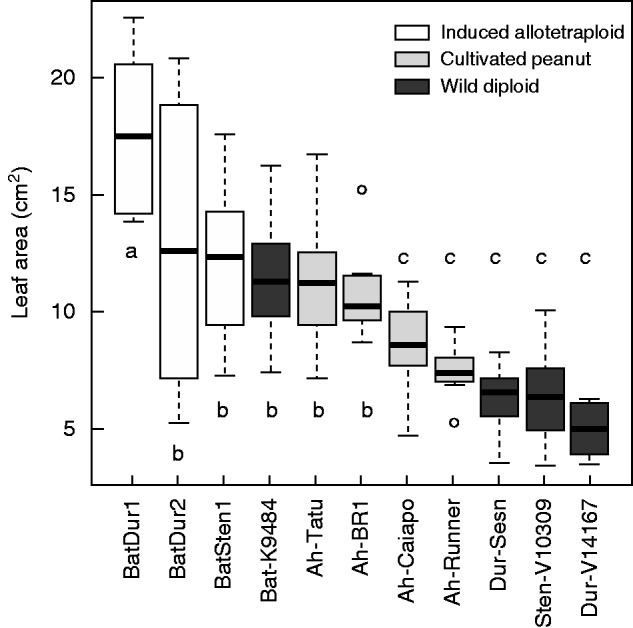
Box plot diagram showing the area of the first expanded leaf of wild diploids, cultivated peanut and induced allotetraploids. Boxes with same letters do not differ significantly at *P* < 0·05. The box contains 50 % of the data points. Bars across boxes represent the median. The top and bottom ends of the ‘whiskers’ represent the highest and lowest values observed. Circles represent outliers.

**Fig. 3.**
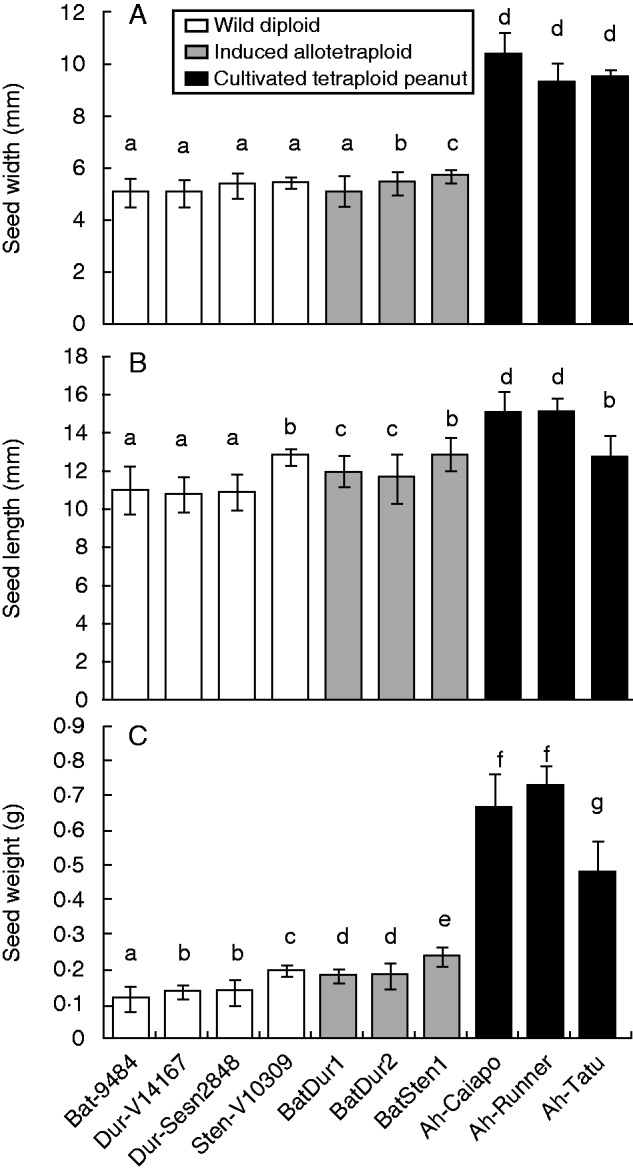
Seed dimensions: (A) width, (B) length and (C) weight of wild diploids, induced allotetraploids and cultivated tetraploid peanut (as indicated in the key). Bars with the same letters do not differ significantly at *P* < 0·05.

Diploid plants tend to have smaller leaves than tetraploid peanut. *Arachis batizocoi* is an exception, having leaves larger than any cultivated peanut measured here (Fig. 2). All adult induced allotetraploid plants, like their diploid parents, had trailing growth habit, but with larger overall size, larger leaves (Fig. 2) and greater vigour. The allotetraploids produced seed abundantly (ranging between 40·6 and 68·2 seeds per plant on average), in numbers comparable with those of their parental wild species (45·8−73·9), and with those of cultivated peanut (44·2−66·5), and did not differ among themselves. The likelihood ratio determined through generalized linear model Poisson was 7·457 and the *P*-value was 0·024. For the induced allotetraploids, weight, length and width of the seeds produced were slightly larger than those of wild parents, and lower than those of cultivated peanut (Kruskal–Wallis, *P* < 0·05). The exception is the allotetraploid BatSten1, whose seed length was comparable with that of IAC-Tatu, a Valencia peanut type (Fig. 3).

### Hybridization of *A. hypogaea* with the induced allotetraploids

Crosses were performed between five *A. hypogaea* cultivars belonging to two subspecies, *hypogaea* and *fastigiata*, and the induced allotetraploids (Table 1). Allotetraploids were used as the male parents. Pollinations carried out in the initial 18 d after the beginning of flowering generated high numbers of pegs and seeds, and these decreased considerably after that. Of a total of 3319 flowers pollinated, 234 pegs were produced, from which 133 seeds were obtained (Table 4). Peg production per pollinated flower ranged between 0·033 and 0·112. Seed production per pollinated flower ranged from 0·016 to 0·083 and showed no correlation with peanut variety or subspecies used. However, BatSten1 was the most crossable induced allotetraploid: it crossed with all cultivars of *A. hypogaea*, and produced the highest rates of pegs per pollinated flower and of seed production per pollinated flower (average of 0·0978; Table 4).

**Table 4.** Crossability of hybrids between peanut cultivars and induced allotetraploids

Female parent		Male parent	No. of pollinated flowers	No. of pegs	No. of seeds	No. of pegs/pollinated flower	No. of seeds/pollinated flower	No. (%) of fertile *F*_1_ plants	Average no. of seeds/fertile *F*_1_ plant
Cultivar	Subspecies								
IAC-Caiapó	*hypogaa*	BatDur1	292	24	8	0·082	0·027	6 (85·7)	5·2
		BatDur2	255	17	8	0·067	0·031	12 (92·3)	9·8
		BatSten1	166	18	10	0·108	0·060	13 (56·5)	6·0
Runner IAC 886	*hypogaea*	BatDur1	245	8	4	0·033	0·016	4 (100·0)	27·3
		BatDur2	264	13	9	0·049	0·034	11 (100·0)	12·3
		BatSten1	169	14	7	0·083	0·041	8 (61·5)	1·0
55-437 (Senegal)	*fastigiata*	BatDur1	313	16	7	0·048	0·022	12 (100·0)	3·8
		BatDur2	277	22	16	0·079	0·058	20 (100·0)	1·2
		BatSten1	217	18	13	0·083	0·060	1 (3·3)	4·0
BRS-Havana	*fastigiata*	BatDur1	202	14	7	0·069	0·035	10 (100·0)	1·8
		BatDur2	185	14	10	0·076	0·054	10 (90·9)	3·7
		BatSten1	145	15	11	0·103	0·083	0 (0·0)	N/A
BR1	*fastigiata*	BatDur1	220	11	7	0·050	0·032	7 (100·0)	0·4
		BatDur2	208	12	7	0·058	0·034	2 (25·0)	0·0
		BatSten1	161	18	9	0·112	0·056	0 (0·0)	N/A
Total			3319	234	133				

N/A, not applicable.

*F*_1_ hybrids between cultivated peanut and induced allotetraploids had variable fertility, evaluated as the production of *F*_2_ seeds. Most hybrid combinations produced a mixture of fertile and sterile *F*_1_ plants (Table 4). Fertility varied according to cultivated peanut × allotetraploid combination. The percentage of fertile *F*_1_ plants from allotetraploids originating from *A*. *duranensis* (BatDur1 or BatDur2) was generally very high (>85 %), with the exception of *A. hypogaea* ‘BR1’ × BatDur2 (25 % fertile). On the other hand, the percentage of fertile *F*_1_ plants derived from the allotetraploid originating from *A*. *stenosperma* (BatSten1) was lower, with cultivars of the subspecies *hypogaea* ranging from 56·5 to 61·5 %, while with the subspecies *fastigiata* ranged from 0 to 3·3 % (Table 4). Also, the subspecies of cultivated peanut had a large influence on the number of seeds produced per fertile *F*_1_ for the three allotetraploids, being higher from crosses derived from cultivars of ssp*. hypogaea* than from those derived from ssp*. fastigiata*. Notably, during the period of data collection for this study, the F_1_ hybrids initially observed as being sterile remained sterile, but, in subsequent seasons, a number of them spontaneously became fertile. It was also noted that some *F*_1_ combinations involving ssp. *fastigiata* displayed unexpected subtle phenotypic variability.

**Fig. 4.**
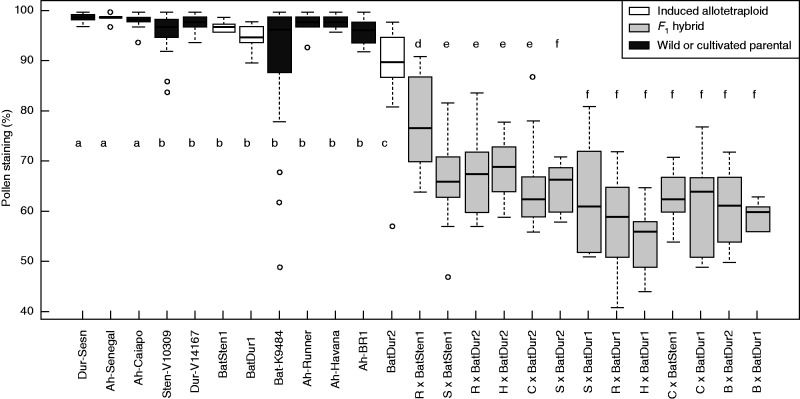
Estimated pollen viability of wild accessions, peanut cultivars, induced allotetraploids and *F*_1_ hybrids between peanut cultivars and induced allotetraploids, estimated by percentage staining. On the *x*-axis, B = *A. hypogaea* ‘BR1’, C = *A. hypogaea* ‘IAC-Caiapo’, H* = A. hypogaea* ‘BRS Havana’, R = *A. hypogaea* ‘IAC Runner 886’, S = *A. hypogaea* ‘55437’ (Senegal). *F*_1_ hybrids are hybrids between cultivated peanut and induced allotetraploids. Bars with the same letters do not differ significantly at *P* < 0·05. The box contains 50 % of the data points. Bars across boxes represent the median. The top and bottom ends of the ‘whiskers’ represent the highest and lowest values observed. Circles represent outliers.

Estimated pollen viability using acetic carmine staining of wild parents, cultivated peanut and induced allotetraploids plants ranged from 89·7 to 98·8 %, and showed little variation regardless of the accession, cultivar or allotetraploid (χ^2 ^= 2637·1, d.f. = 24, *P* < 0·001) (Fig. 4). These results suggest that estimated pollen viability is not the major contributor to differences in success of *F*_1_ production.

Pollen viability was also estimated to investigate the difference in fertility of *F*_1_ hybrids. All hybrid plants derived from cultivated peanut × induced allotetraploids showed much lower levels of estimated pollen viability (average of 64 %) than their parents (average of 98 % for cultivated peanut and 93·3 % for allotetraploids), and this was not dependent on the peanut subspecies used as female parent or on the allotetraploid used as male parent (Fig. 4).

For the purpose of comparison with *A. hypogaea* ‘Runner IAC 886’ × [*A. batizocoi* K9484 × *A. duranensis* V14167]^4×^, the estimated pollen viability of a previously obtained cultivated peanut × induced allotetraploid F_1_ hybrid was also determined. This hybrid was the *A. hypogaea* ‘Runner IAC 886’ crossed with [*A. ipaensis* KG30076 × *A. duranensis* V14167]^4×^ (Fávero *et al*., 2006). This allowed the direct comparison of the influence on fertility of *A. batizocoi* compared with *A. ipaensis*, the species now considered the most probable B genome donor to cultivated peanut (Seijo *et al.*, 2004, 2007; Moretzsohn *et al.*, 2013), in hybrids involving cultivated peanut. The average pollen viability of *A. batizocoi*-derived hybrids was 77·5 %, whereas for *A. ipaënsis*-derived hybrids the average viability was 82·3 %. There was no significant difference in pollen viability between these two hybrids (*P* < 0·05).

### Cytogenetic analyses

**Fig. 5.**
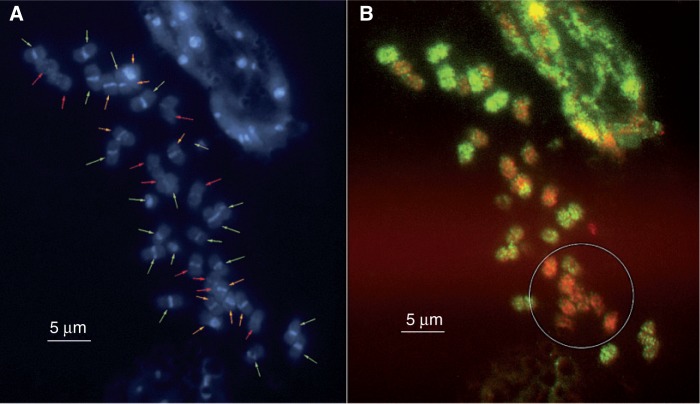
Cytogenetic analysis of the hybrid between *A. hypogaea* ‘55437’ and (*A. batizocoi* K9484 × *A. stenosperna* V10309) using GISH. Metaphase spreads of the hybrid were probed with a mixture of dig-labelled total genomic DNA from *A. duranensis* V14167 and biotin-labelled total genomic DNA from *A. ipaënsis*. (A) DAPI counterstain showing 40 chromosomes, 30 of which had strong centromeric DAPI bands, typical for *Arachis* A genome and *A. batizocoi* chromosomes. The coloured arrows indicate the nature of the individual chromosomes as derived from the combination of DAPI banding and GISH results (see text): green arrows, *A. duranensis* or *A. stenosperma*; red arrows, *A. ipaënsis*; orange arrows, *A. batizocoi*. (B) Superimposed image made from the individual FITC and Cy3 channels after GISH showing 20 green chromosomes that hybridized with the *A. duranensis* A genome probe and 20 red-orange chromosomes from hybridization with the *A. ipaensis* B genome probe. The circle comprises nine red-orange chromosomes, comprised of four *A. ipaënsis* and five *A. batizocoi* chromosomes. Comparison of hybridization intensities shows that the *A. ipaënsis* probe hybridized slightly more weakly to the *A. batizocoi* chromosomes.

The genomic composition of the hybrid derived from the cross between *A. hypogaea* 55437 and the allotetraploid *A. batizocoi* ‘K9484’ × *A. stenosperma* V10309 was analysed by GISH using total genomic DNA from *A. duranensis* V14167 (A genome, digoxigenin-labelled, green fluorescence) and *A. ipaënsis* KG30076 (B genome, biotin-labelled, red fluorescence) as probes. The expected chromosome composition was 20 chromosomes from the A genome (ten from *A. hypogaea* sub-genome A and ten from *A. stenosperma*), ten from *A. hypogaea* sub-genome B chromosomes and ten chromosomes of the K genome of *A. batizocoi* (Fig. 5A). In order to facilitate interpretation of the results, the images captured from the two fluorescent channels were overlaid (Fig. 5B). The hybrid had 40 chromosomes, of which 20 chromosomes showed green signal (from the *A. duranensis* A genome probe) and 20 showed red-orange fluorescence (from the *A. ipaënsis* B genome probe). In comparsion with the karyotype of DAPI-stained *A. hypogaea* metaphases, where 20 chromosomes of the A genome exhibit clear heterochromatic bands and 20 B genome chromosomes do not, here only ten chromosomes had no or weak centromeric DAPI bands per centromere (Fig. 5A). Comparison with the GISH image (Fig. 5B) shows that among the 20 chromosomes that have hybridized preferentially with the *A. ipaënsis* probe (red signals), ten chromosomes were DAPI banded. This indicates that these chromosomes are derived from *A. batizocoi*, which is characterized by the presence of heterochromatic DAPI bands (Seijo *et al.*, 2004). In addition, *A. batizocoi* shares a sufficient amount of repetitive DNA with the B genome to allow preferential hybridization with the *A. ipaënsis* probe. A comparison of red and orange chromosomes in close proximity (see the circle in Fig. 5B) shows that the intensity of signals is slightly weaker on *A. batizocoi* than on *A. ipaënsis* chromosomes. This observation corroborates results described earlier by Seijo and co-workers (2007), who used *A. batizocoi* and *A. ipaënsis* probes for hybridization of *A. hypogaea* metaphases. Based on the GISH hybridization, coloured arrows were added to the image of the DAPI-stained metaphase (Fig. 5A), assigning the individual chromosomes to the species that contribute to the complex hybrid (green arrows, *A. duranensis* and *A. stenosperma*; red arrows, *A. ipaënsis*; orange arrows, *A. batizocoi*).

### Phylogenetic analysis of A, B and K genomes

The 16 combined TOGs yielded an aligned matrix of 9093 characters, to which were appended 74 characters from simple indel coding. Of the total characters, 8683 were constant; 289 variable characters were parsimony-uninformative and 195 were parsimony-informative. Furthermore, the matrix maximum parsimony homoplasy index was low [0·0679; PAUP* version 4.0b10 (Swofford, 2003)]. In the Bayesian analysis, the distribution of tree log likelihoods of both MrBayes runs was normal, with means of −15 706·3 and −15 705·9, respectively.

**Fig. 6.**
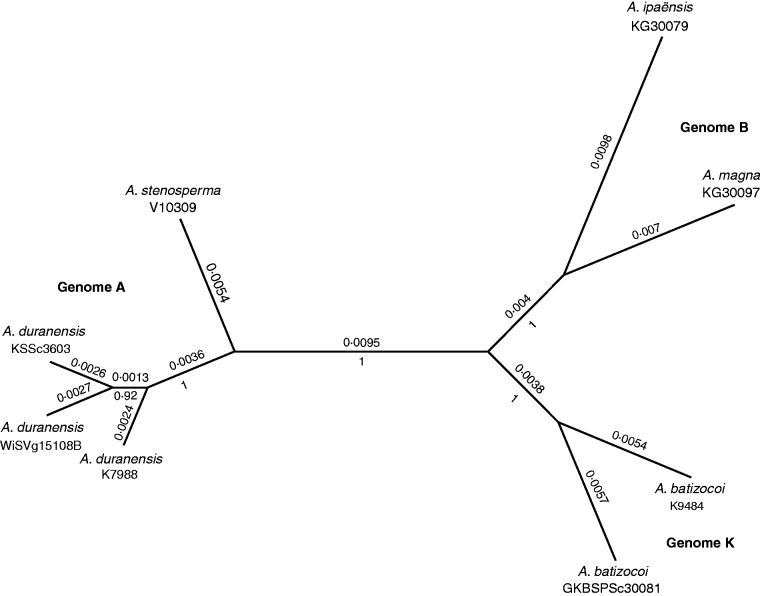
Unrooted Bayesian majority rule tree inferred from 16 combined TOGs with simple indel coding. Branch lengths are given above the branches and Bayesian posterior probabilities are given below. For each genotype, the species name and accession number are given.

The two K genome *A. batizocoi* accessions included in the analysis clustered together, and were more closely related to *A. magna* and then to *A. ipaënsis*, both B genome species, than to the A genome species, *A. stenosperma* and *A. duranensis* (Fig. 6). With the exception of one branch within the three *A. duranensis* accessions, all branches within the Bayesian consensus phylogram possessed posterior probabilities of 1·0.

#### Testing for ancient hybridization events in *Arachis* species evolution.

To test the possibility that *A. batizocoi* is derived from an ancient hybridization event between A and B lineages, and reticulate evolution, some statistical analyses were performed. The phi test for evidence of recombination among the included diploid *Arachis* species was not significant (*P* = 0·1408; 114 informative sites; mean = 0·126; variance = 3·354E-4; observed = 0·106) for the matrix comprising the concatenation of 16 TOGs and encoded indel characters from *A. batizocoi* (GKBSPSc30081), *A. duranensis* (K7988), *A. ipaënsis*, *A. magna* and *A. stenosperma*. In contrast, when *A. hypogaea* was included in the data matrix, the phi test was highly significant (*P* = 5·273E-7; 148 informative sites; mean = 0·254; variance = 4·852E-4; observed = 0·147), reflecting the allotetraploid nature of this species, most probably comprising the A genome from *A. duranensis* and the B genome from *A. ipaënsis.* The parsimony splits analysis detected negligible evidence for reticulate evolution among the diploid *Arachis* species, whereas inclusion of *A. hypogaea* in the data set produced the expected phylogenetic network. The lack of evidence of recombination among the included diploid *Arachis* species was confirmed in the DualBrothers analysis, where a single topology predominated over the entire alignment.

## DISCUSSION

### The K genome is closer to the B than the A genome: phylogenetic, GISH and genetic evidence

*Arachis batizocoi* has a long and interesting history in *Arachis* phylogenetic studies and of utilization in peanut breeding. Initially thought of as the B genome donor to *A. hypogaea* (Husted, 1936; Smartt *et al.*, 1978; Stalker *et al.*, 1991; Singh and Smartt, 1998), its genome was subsequently shown to be divergent from the B genome and was classified in a separate genome type, the ‘K’ genome (Robledo and Seijo, 2010). Since this study, the phylogenetic placement of the K genome relative to the A and B genomes has been a matter of debate. Using internal transcribed spacer (ITS) and plastid regions, Friend and co-workers (2010) found that *A. batizocoi* clustered with the *s.s.* B genome species. Similar results were obtained by Bechara and co-workers (2010), using ITS and 5.8S rDNA sequences. The analysis of Grabiele and co-workers (2012) using chloroplast DNA (cpDNA) also placed it with the B genome *s.s.* species, but basally diverging. In contrast, fluorescent *in situ* hybridization (FISH) mapping of rDNA loci has shown that K genome species are more closely related to A genome species (Robledo and Seijo, 2010).

To resolve these conflicting results, we used only a few representatives from each genome type, but, concentrating on these, we were able to use a larger data set. This consisted of 16 different intron sequences, all from different genes spread over diverse genome locations. The analysis of this data set yielded a reconstructed phylogeny of very high confidence where all the branches that define the genome types have posterior probabilities of 1·0 (Fig, 6). The tree shows that while *A. batizocoi* is distinct, it is definitively more closely related to the B than to the A genome species. Furthermore, this conclusion was supported by results from GISH. The use of *A. ipaensis* and *A. duranensis* genomic DNAs as probes on the chromosomes of a three-way intragenomic hybrid *A. hypogaea* × [*A. batizocoi* × *A. stenosperma*]^4×^ allowed the direct comparison of signals from the different component genomes on the same chromosomes set. This clearly indicated a greater affinity of the *A. batizocoi* genome for *A. ipaensis* (B genome) than for *A. duranensis* (A genome), implying a greater overall similarity of repetitive DNA content of B and K genomes. This is also consistent with genetic data that show that the genome of *A. batizocoi* recombines with the B genome of cultivated peanut in a remarkably normal way. Finally, to test the possibility that *A. batizocoi* is derived from an ancient hybridization event between A and B lineages, we tested for reticulate evolution and recombination. Using three tests, we found negligible evidence for this. We consider that these multiple lines of evidence present a significant result that effectively resolves the uncertainties of the principle phylogenetic affinities of *A. batizocoi.*

### The K genome has high crossability, and derived induced allotetraploids show the gigas effect

The *A. batizocoi* accession used here showed high crossability with the A genome accessions. In our experience, using the same A genome accessions, it was substantially easier to obtain primary diploid hybrids using *A. batizocoi* than *A. ipaensis* (results not shown). Highly infertile diploid hybrids were produced with all seven diploid K × A crossing combinations performed here. All individuals of two hybrid combinations exhibited symptoms typical of hybrid incompatibility, and one spontaneously died. The other six were treated with colchicine. In spite of similar efforts made to induce polyploids with these six, only three produced allotetraploids. In all of these three combinations, multiple independent allopolyploidy events were obtained. These results indicate that certain genome combinations are more crossable, certain combinations are more amenable to producing hybrid plants with normal vegetative growth, and certain combinations are more pre-disposed to induction of polyploidy. This is consistent with the barriers between species occurring at different levels (Stace, 1989). The induced allotetraploids produced were all phenotypically uniform, vigorous and highly fertile. Only immediately after germination did they show developmental irregularities. Some primary leaves of most seedlings were bleached and morphologically altered, but these abnormalities disappeared after a few weeks. The appearance of these morphological abnormalities has continued in the seedling phase for several generations, but with an overall tendency to reduce. The induced allotetraploids all showed the characteristic gigas effect (Acquaah, 2012). Being ‘doubled interpecific hybrids’, they have increased chromosome complements leading to gigantism, especially in leaf and overall plant size. They also show a permanent fixation of heterozygosity and hybrid vigour. Therefore, while they combine the genomes of the two parental wild species, they also display new phenotypes because of their ploidy (Leal-Bertioli *et al.*, 2012).

### Compatibility of the A genomes ‘rescues’ the cultivated peanut × induced allotetraploid hybrids from the incompatibility of the B and K genomes

*Arachis duranensis* is the most likely ancestor of the A genome of *A. hypogaea*, and has good genetic compatibility with it when used as one of the parents in allotetraploids (Seijo *et al.*, 2007; Fonceka *et al.*, 2012). *Arachis stenosperma* produces hybrids with *A. duranensis* that, although reduced in fertility, were successfully used to produce a set of recombinant inbred lines (Moretzsohn *et al.*, 2005; Leal-Bertioli *et al.*, 2009; Shirasawa *et al.*, 2013). In contrast, the same accession of *A. batizocoi* used here produced highly infertile hybrids when crossed with *A. ipaensis* (Krapovickas and Gregory, 1994), the wild diploid known to be very similar to the B genome of *A. hypogaea*. Also hybrids between cultivated peanut and the amphidiploid TxAG-6 (which have *A. batizocoi* as one of the parents; Simpson *et al.*, 1993) have low fertility, although this was never precisely quantified (C.H. Simpson, Texas A&M, USA, pers. comm.).

Therefore, we anticipated that hybrids between *A. hypogaea* and the induced allotetraploids would combine generally compatible interactions on the A genomes, and generally incompatible interactions between the B and K genomes. Correspondingly, although many *F*_1_ hybrids between *A. hypogaea* and the induced allotetraploids showed fertility, all had lower estimated pollen viability and lower fertility levels than any of the wild or cultivated accessions, or indeed the induced allotetraploids themselves. The *F*_1_ hybrids derived from induced allotetraploids originating from *A. duranensis* showed a distinct tendency of being more fertile than *F*_1_ hybrids derived from the induced allotetraploids originating from *A. stenosperma.* Remarkably, this difference was much greater in hybrids involving *A. hypogaea* ssp. *fastigiata* than in those involving ssp. *hypogaea* cultivars. This indicates a notable genetic divergence between the two subspecies of cultivated peanut, a divergence that seems incongruent with the low levels of DNA polymorphism found between them, and the very recent species origin (Bertioli *et al.*, 2011).

Generally, it was evident that the fertility of *F*_1_ hybrids between *A. hypogaea* and the induced allotetraploids was more influenced by the compatible genome interactions than the incompatible interactions. In other words, the compatibility of the A genome species in the *A. hypogaea* × induced allotetraploid *F*_1_ hybrids ‘rescued’ them from the consequences of the incompatible B–K interactions. This was particularly emphasized by observation that the estimated pollen viabilities of *A. hypogaea* ‘Runner IAC 886’ × [*A. batizocoi* K9484 × *A. duranensis* V14167]^4×^ and *A. hypogaea* ‘Runner IAC 886’ × [*A. ipaensis* KG30076 × *A. duranensis* V14167]^4×^ were not significantly different. This observation suggests that induced allotetraploids made for peanut breeding should use at least one species that is highly related to one of the component sub-genomes of cultivated peanut. The use of two divergent species will probably produce fertile induced allotetraploids, but these will form more sterile hybrids with *A. hypogaea*.

### The variability in fertility between different *F*_1_ hybrids from the same parents is difficult to explain

The variability in fertility observed between different *F*_1_ hybrid individuals resulting from the exact same combination of *A. hypogaea* and induced allotetraploid genomes is intriguing. In all cases, the exact same combination produced some fertile and some infertile hybrids. *Arachis hypogaea*, being highly autogamous, is highly homozygous, and the induced allotetraploids should be completely so. Therefore, we would expect their *F*_1_ progeny to be very uniform, but they definitively were not so. Intriguingly, some of the infertile *F*_1_ individuals regained fertility in subsequent seasons. We believe that plausible explanations for these observations may lie in stochastic events following hybridization possibly involving activation of transposons, DNA methylation or other genome instability.

### Concluding remarks

In conclusion we show here that the K genome of *A. batizocoi* is distinct from the A and B *Arachis* genomes, but that it is more closely related to the B genome. The combination of high crossability, divergent genome and, when incorporated in an induced allotetraploid, fertility when crossed with cultivated peanut makes *A. batizocoi* in itself an attractive source of new alleles for improving the peanut crop, especially in crosses involving ssp. *hypogaea.* However, when the main interest is introgression of wild A genome alleles, incorporation of the A genome species in an allotetraploid with a classic B genome species will probably lead to more fertile progeny.
